# Angiographic characteristics of culprit lesions in infarct related artery and correlation of TIMI score with SYNTAX score to predict extent and severity of coronary artery disease in patients undergoing primary percutaneous coronary interventions

**DOI:** 10.12669/pjms.40.1.7750

**Published:** 2024

**Authors:** Rizwanullah, Jawaid Akbar Sial, Nasir Ali, M. Shehzad

**Affiliations:** 1 Rizwanullah, FCPS Post Fellow Interventional Cardiology, National Institute of Cardiovascular Diseases, Karachi, Pakistan; 2 Jawaid Akbar Sial, FCPS Professor of Cardiology, National Institute of Cardiovascular Diseases, Karachi, Pakistan; 3 Nasir Ali, FCPS Post Fellow Interventional Cardiology, National Institute of Cardiovascular Diseases, Karachi, Pakistan; 4 Muhammad Shehzad, FCPS Post Graduate Trainee, National Institute of Cardiovascular Diseases, Karachi, Pakistan

**Keywords:** CAD, STEMI, TIMI risk score, SYNTAX score, PPCI

## Abstract

**Objective:**

The current study was designed to explore the relationship of TIMI and SYNTAX risk score to predict the CAD extent and severity in STEMI patients.

**Methods:**

For this cross-sectional study, 304 STEMI patients undergoing PPCI were enrolled at Department of Interventional Cardiology NICVD Karachi from September 2021 to January 2022. and the TIMI risk score was determined at enrolment. Based on these scorings, the patients were grouped as low, intermediate, and high risk, i.e., a score of ≤ 3, 4 to 7, and ≥ 8, respectively. The SYNTAX scores were utilized to assess the extent of CAD.

**Results:**

Statistically significant difference was found in symptoms to balloon time (p=0.001), history of diabetes (p=0.006), angina (p=0.011), obesity (p=0.048), STEMI type (p=0.003), Killip classes (p=0.000), Infarct-Related Artery (p=0.006), number of diseased vessels (p<0.01), LMS > 50% (p=0.000), PCI type (p<0.01), collateral circulation (p<0.01), In-hospital mortality (p<0.01), LV support (p<0.01), and post-procedural TIMI flow (p=0.013), among the three TIMI risk groups. Significant correlation was found among TIMI risk score and SYNTAX score.

**Conclusion:**

It is observed that the TIMI risk scores are highly correlated with the SYNTAX Score in predicting the CAD severity in STEMI patients.

## INTRODUCTION

Cardiovascular disease is the main cause of mortality globally, according to the WHO’s list of the top causes of death and disability.^1^ One of the three major epicardial coronary arteries is completely occluded in STEMI and the preferred course of treatment includes mechanical opening via PPCI.^2^

The patency in terms of TIMI flow grade and culprit lesion’s complexity of the infarct related artery (IRA) determines the infarct size and procedural outcome in patients with STEMI who qualify for PPCI according to contemporary guidelines.^3^ According to the results of a recent study, 26% of PPCI patients had spontaneous reperfusion with TIMI Grade-3 flow in IRA on coronary angiogram. Seventy two percent of such patients had type-A lesions compared to 32% in IRAs having TIMI flow grade of ≤ 2, with statistical significance.^4^

The clinical TIMI score is a quick and accurate bedside tool for predicting thirty-day mortality and MACE in STEMI patients. TIME-II trial investigators first developed this tool in 2000 as the mathematical sum of risk scores assigned to individual mortality predictors, which was subsequently validated in numerous trials.^5^ This scoring system, however, does not include the angiographic findings nor tells about the intricacy and severity of the anatomy of the CAD. On the other hand, a validated risk scoring system (SYNTAX score) is used to predict MACE after invasive imaging of the coronary arterial tree.^6^

By investigating the relationship between clinical TIMI scores and angiographic signs of the severity of coronary artery disease (CAD) in ST-Elevation Myocardial Infarction (STEMI) patients who get primary percutaneous coronary intervention (PPCI), this study addresses a research gap. In Non-ST-Elevation ACS (NSTE-ACS) patients, studies have shown a good link between TIMI scores and angiographic results; 7,8 however, there is a paucity of information on STEMI patients both domestically and abroad.^9^ Therefore, the purpose of this study was to explore the possibility of a correlation between CAD severity and TIMI risk scores in STEMI patients undergoing PPCI. The research seeks to improve risk assessment and therapy selection for STEMI patients, resulting in better clinical care and prognosis evaluation in this crucial population.

## METHODS

NICVD’s department of Interventional cardiology Karachi, undertook this single-centre, prospective cross-sectional study from September 2021 to January 2022. Patients as per the criteria for inclusion were enrolled after providing written consent. With an expected strength of positive correlation between the SYNTAX and the TIMI risk score of 0.16^9^, with a significance level of 0.05 and test power of 80%, the calculated sample size was n= 304.

### Ethical Approval

The Hospital Ethical and Research Committee granted ethical permission (Reference# ERC-96/2021; Dated 21 September 2021).

STEMI patients of age above 18 years, ST elevation ≥ 1mm (limb leads) and ≥ 2mm (precordial leads) and within guidelines recommended time window for PPCI, were considered eligible for inclusion. At the same time, patients with pre-existing LBBB, Prior MI, PCI, and CABG were kept in the exclusion criteria.

Demographic variables, risk factors, vital signs, weight/height, and clinical examination findings of the chest and precordium were documented on a designed proforma. Twelve lead standard ECGs were obtained in ER to look for ST elevations. TIMI score was calculated from the documented clinical variables, including age, Diabetes/HTN/Angina history, SBP, Heart Rate, Killip class, Anterior STE/LBBB, Weight < 67 kg, and Time to treatment > 4 hours as first-time developed by Morrow et al.^5^ Based on the TIMI risk score, patients having a TIMI score ≤ 3 were considered to be at low risk, those with TIMI score four to seven had intermediate risk, while those with a score ≥ 8 were at high risk. Patients were shifted to the catheterization Lab for PPCI, and two experienced cardiologists assessed their angiograms for segmental visual analysis of CAD and culprit lesion characteristics in IRA as per operational definition (Appendix I). SYNTAX scores were calculated after the assessment of angiograms.

### Statistical analysis

The statistical analysis was conducted using SPSS Ver. 22.0. The descriptive statistics were used to display the continuous and categorical variables. To compare categorical variables between the groups, the Chi-square test and Fischer’s exact test were utilized. While one-way ANOVA was applied to continuous variables. Using Pearson’s correlation, the relation between the TIMI and SYNTAX risk scores was explored.

## RESULTS

Among the TIMI Risk groups mean age was higher (p<0.01) in intermediate and high-risk groups compared to the low risk group as shown in Table-I. The prevalence of Diabetes and past history of angina was significantly more common in high risk TIMI group. The mean for symptoms to balloon time was significantly higher in high risk TIMI group.

**Table-I:** Patient’s demographic characteristics by TIMI Risk group.

Variables		TIMI Risk groups			p-value

		Low (n=174)	Intermediate (n=110)	High (n=20)	
Age (years); mean±SD		53.2±9.26	61.72±10.60	63.5±10.41	0.000*
Symptoms to balloon time (min); mean±SD		258.48±135.85	321.93±158.17	321±167.70	0.001*
Gender; n(%)	Male	143(82.2)	91(82.7)	17(85.0)	0.950
	Female	31(17.8)	19(17.3)	3(15.0)	
Risk Factors; n(%)	DM	58(33.3)	54(49.1)	12(60.0)	0.006*
	HTN	101(58.0)	73(66.4)	16(80.0)	0.091
	Smoking	81(46.6)	48(43.6)	8(40.0)	0.797
	Angina	40(23.00)	41(37.30)	9(45.00)	0.011*
	CRF/CKD	2(1.1)	-	-	0.471
	Obesity	39(22.40)	13(11.80)	2(10.00)	0.048*
Family History of CAD; n(%)		74(42.5)	34(30.9)	8(40.0)	0.143
STEMI Type; n(%)	IWMI	93(53.4)	39(35.5)	4(20.0)	0.003*
	AWMI	77(44.3)	70(63.6)	15(75.0)	
	LWMI	4(2.3)	1(0.9)	-	
Killip on admission; n(%)	I-II	171(98.8)	105(95.5)	11(55.0)	0.000*
	III-IV	2(1.2)	5(4.5)	9(45.0)	

* p<0.05 is considered significant.

The comparison of procedural characteristics is shown in Table-II. The distribution of arteries involved in infarcts varied significantly between TIMI Risk groups (p=0.006). The TIMI Risk groups showed significantly different in-hospital mortality (p<0.01). The predictive usefulness of TIMI risk scores in foretelling unfavourable outcomes in STEMI patients was highlighted by the strikingly greater mortality rate of the High-risk group compared to the Low and Intermediate risk groups. The TIMI Risk groups differed significantly in their need for left ventricular (LV) assistance (p<0.01). The TIMI Risk groups exhibited significant differences in SYNTAX scores (p<0.01). It was confirmed that there is a direct correlation between clinical risk assessment and the severity of CAD in higher TIMI risk groups as they were associated with higher SYNTAX scores. The connection between the TIMI risk score and the SYNTAX score was substantial and positive (r = 0.681, p<0.01).

**Table-II:** Baseline angiographic and post-procedural characteristics by TIMI Risk Score groups.

Variables		TIMI Risk Index groups			p-value

		Low (n=174)	Intermediate (n=110)	High (n=20)	
IRA	LAD	79(45.4)	70 (63.6)	15(75.0)	0.006*
	LCx	19(10.9)	8(7.3)	1(5.0)	
	LMS	-	1(0.9)	1(5.0)	
	RCA	74(42.5)	30(27.3)	3(15.0)	
	RI	2(1.1)	1(0.9)	-	
Lesion type	A	23(13.20)	12(10.9)	1(5.0)	0.602
	B2	6(3.40)	2(1.80)	-	
	C	145(83.3)	96(87.3)	19(95.0)	
Number of vessels diseased.	One	72(41.4)	21(19.1)	5(25.0)	<0.01*
	Two	64(36.8)	35(31.8)	4(20.0)	
	Three	38(21.8)	54(49.1)	11(55.0)	
Dominance	Co-dominant	2(1.10)	2(1.80)	-	0.951
	Right	154(88.50)	95(86.40)	18(90.00)	
	Left	18(10.30)	13(11.80)	2(10.00)	
LMS > 50%	Yes	2(1.10)	6(5.50)	11(55.0)	<0.01*
	No	172(98.90)	104(94.50)	9(45.00)	
PCI type	DES	170(97.70)	100(90.90)	18(90.00)	0.027*
	POBA	4(2.30)	10(9.10)	2(10.0)	
Pre-procedural TIMI flow grade	0	93(53.4)	65(59.1)	17(85.0)	0.242
	I	33(19.0)	20(18.2)	2(10.0)	
	II	42(24.1)	22(20.0)	1(5.0)	
	III	6(3.4)	3(2.7)	-	
Thrombus grade IRA	0	1(0.60)	-	-	0.448
	1	3(1.7)	2(1.8)	-	
	2	26(14.9)	16(14.50)	-	
	3	20(11.5)	15(13.6)	2(10.0)	
	4	31(17.8)	11(10.0)	2(10.0)	
	5	93(53.4)	66(60.0)	16(80.0)
Collateral Circulation		16(9.2)	22(20.0)	12(60.0)	<0.01*
In-hospital mortality		3(1.7)	11(10.0)	14(70.0)	<0.01*
LV support		16(9.2)	24(21.8)	18(90.0)	<0.01*
Post-procedural TIMI flow	II	15(8.6)	11(10.0)	6(30.0)	0.013*
	III	159(91.4)	99(90.0)	14(70.0)	
Risk scores	SYNTAX score	18.77±5.36	26.73±4.72	34.75±5.78	<0.01*
	TIMI score	2.06±0.87	4.83±0.93	9.00±0.97	<0.01*

* p<0.05 is considered significant.

## DISCUSSION

The TIMI risk score was initially developed with clinical endpoints like death, myocardial infarction, or urgent revascularization in mind. This study was aimed to determine the degree to which risk-scoring systems and the severity of CAD are associated. The severity and extent of CAD are assessed using various risk scores like the TIMI risk score, SYNTAX score, and Gensini scores. Even though SYNTAX and Gensini scoring systems are recognized validated predictors of unfavourable CAD events and have benefits, one drawback is that they must be used in conjunction with invasive procedures like coronary angiography to assess the CAD severity.^10^-^12^ Therefore, a simple, affordable, and non-invasive risk stratification method must be used to ascertain the CAD severity. A recognized predictor of unfavourable CAD events in CVD patients is the GRS.^13^ The GRS does not assess the complexity and morphology of coronary lesions or the CAD severity.

Studies using the GRS and TIMI risk score (TRS) revealed that the three most crucial variables predicting mortality are SBP, heart rate, and age.^14^ Morrow et al., proved that TRI, an index made up of these three parameters, can be used to determine the ris*k of* ACS patients.^9^ In a different study, Truong and others showed that in STEMI patients, TRI accurately predicts mortality and heart failure.^15^

In the present study of 304 patients, 57.2% were at low risk (TIMI ≤ 3), 36.2% were at moderate risk (TIMI 4 to 7), and 6.6% were at high risk (TIMI ≥ 8). Similarly, in a study by Talreja et al., including 150 patients, 52.7% of the cases were classified as high risk, and 47.3% were at moderate risk.^16^ In a study by Iltaf and colleagues, 31.84% of the patients receiving primary PCI had a TIMI risk score of ≥ 9.^17^ The study also found that patients with a TIMI score of nine had higher rates of adverse events and complications, including death, stroke, and pulmonary edema, compared to the outcomes of primary PCI that had previously been reported in sizable registry-based studies.^18^,^19^

Comparing the TIMI risk scores with number of diseased vessels, 3VD have been shown to be more common in each increased risk category. Similarly, Santos et al. assessed the correlation between risk scores and coronary anatomy among 683 NSTE-ACS patients; it was reported that 3VD or left main coronary disease is more frequent in each increased risk category.^20^ The SVD was more common in patients with TIMI scores of zero to two than in those with scores of five to seven, according to the PRISM-PLUS study.^21^ Irrespective of the TIMI risk score, the frequency of 2VD was the same. Patients in Group-3 with a TIMI score of five to seven were more likely to have 3VD or left main disease than patients in Group-2 with a score of three to four (or patients in Group-1 with a score of zero to two.

In addition to the SYNTAX score, the TIMI risk scores were significantly correlated with the in-hospital mortality rate (r = -0.494, p < 0.01). Furnaz et al. found a strong linear correlation between the TIMI score and mortality rate^22^. With a TIMI score ≤ 5, there was a mortality rate of 5.6%, and with a score ≥ 8, there was a mortality rate of 54.4%. The AUC value of 0.709 (0.591-0.827) was identified as the TIMI score’s predictive value for in-hospital mortality following primary PCI. They further reported that the In-hospital mortality rate was significantly associated with the TIMI score (p<0.001). Among patients with a TIMI score of 0–4, the mortality rate was 3.1%, which raised to 34.6% at the score of eight.

**APPENDIX I-Operational definitions**

- **Infarct Related Artery (IRA)** is defined as the epicardial coronary artery which supplies the myocardium under jeopardy represented by ST segment elevation in corresponding ECG leads.
- **Culprit lesion** is defined as the atherosclerotic segment with narrowest luminal diameter of IRA having variable TIMI flow grade on visual assessment of angiographic films with or without evidence of plaque ulceration. These lesions will be classified into one of the following types according to ACC/AHA classification. Type-A, Type-B1, Type-B2, Type-C - We will categorize Type A and B1 as simple and Type B2 and C complex lesions.
- **TIMI risk score** is defined as the simple arithmetic sum of point score of all eight independent predictors of mortality which include Age, Diabetes/HTN/Angina history, Systolic BP, Heart Rate, Killip class, Anterior STE/LBBB, Weight < 67kg and Time to treatment > 4hrs as first time developed by Morrow et al. Based on the TIMI risk score, patient will be assigned into one of the following groups. TIMI score ≤ 3 = Low risk, TIMI score 4-7 = Intermediate risk, TIMI score ≥ 8 = High risk
- **Extent and severity of CAD** is defined by the Syntax score which is the sum of point score for ≥ 50% luminal narrowing in ≥ 1.5 mm diameter epicardial coronary artery segments. This score will be calculated online at http://www.SYNTAXscore.com.
- **Primary Percutaneous Coronary Intervention** is defined as catheter based therapeutic invasive procedure under fluoroscopic guidance to open the stenosed / occluded IRA by mechanical means in STEMI patients.

### Limitations

There were certain limitations in the present research; firstly, only a small number of patients from a single centre made up the study’s population. Second, because there was a male predominance among the participants in our study, the findings might not apply to groups where women predominate.

## CONCLUSION

As determined by angiography, the severity and scope of CAD were correlated with the TIMI risk scores. TIMI risk scores are significantly correlated to Syntax Scores in estimating the severity and extent of the CAD in STEMI patients. Moreover, TIMI risk scores significantly correlate to the in-hospital mortality rate.

### Authors` Contribution

**R, JAS, NA, and MS,** substantial contributions to conception and design, or acquisition of data, or analysis and interpretation of data, revising article it critically for important intellectual content and final approval of the version to be published.

All authors are in agreement to be accountable for all aspects of the work in ensuring that questions related to the accuracy or integrity of any part of the work are appropriately investigated and resolved.
